# Supplementary material for: Microneurotrophin BNN27 Reduces Astrogliosis and Increases Density of Neurons and Implanted Neural Stem Cell-Derived Cells after Spinal Cord Injury
Source: Biomedicines. 2023 Apr 13;11(4):1170. doi: 10.3390/biomedicines11041170 (PMC10135698; doi:10.3390/biomedicines11041170)
Supplement: Supplementary file 1 [file biomedicines-11-01170-s001.zip › biomedicines-2292506-supplementary.pdf]

Supplementary Materials

Microneurotrophin BNN27 Reduces Astrogliosis and Increases Density of Neurons and Implanted Neural Stem Cell-Derived Cells after Spinal Cord Injury

Konstantina Georgelou, Erasmia-Angeliki Saridaki, Kanelina Karali, Argyri Papagiannaki, Ioannis Charalampopoulos, Achille Gravanis and Dimitrios S. Tzeranis

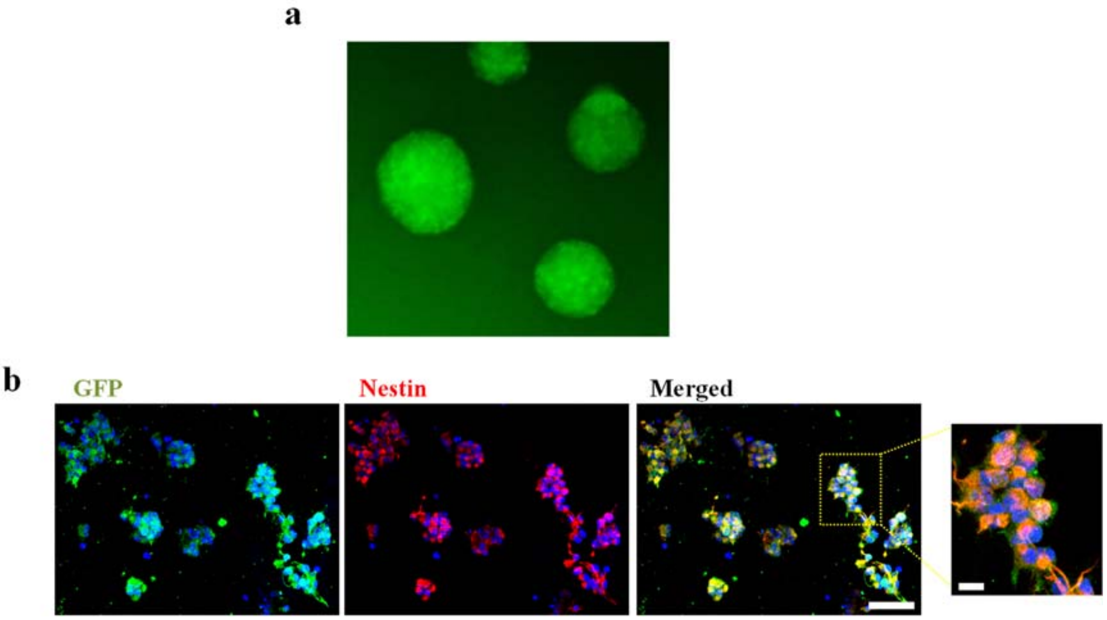

Figure S1. (a) YFP-expressing NSCs in culture as neurospheres. **(b)** YFP-expressing NSCs seeded in PCS for 3 days and immunostained for GFP (green) and Nestin (red). Scale bars: 50 μm and 10 μm.

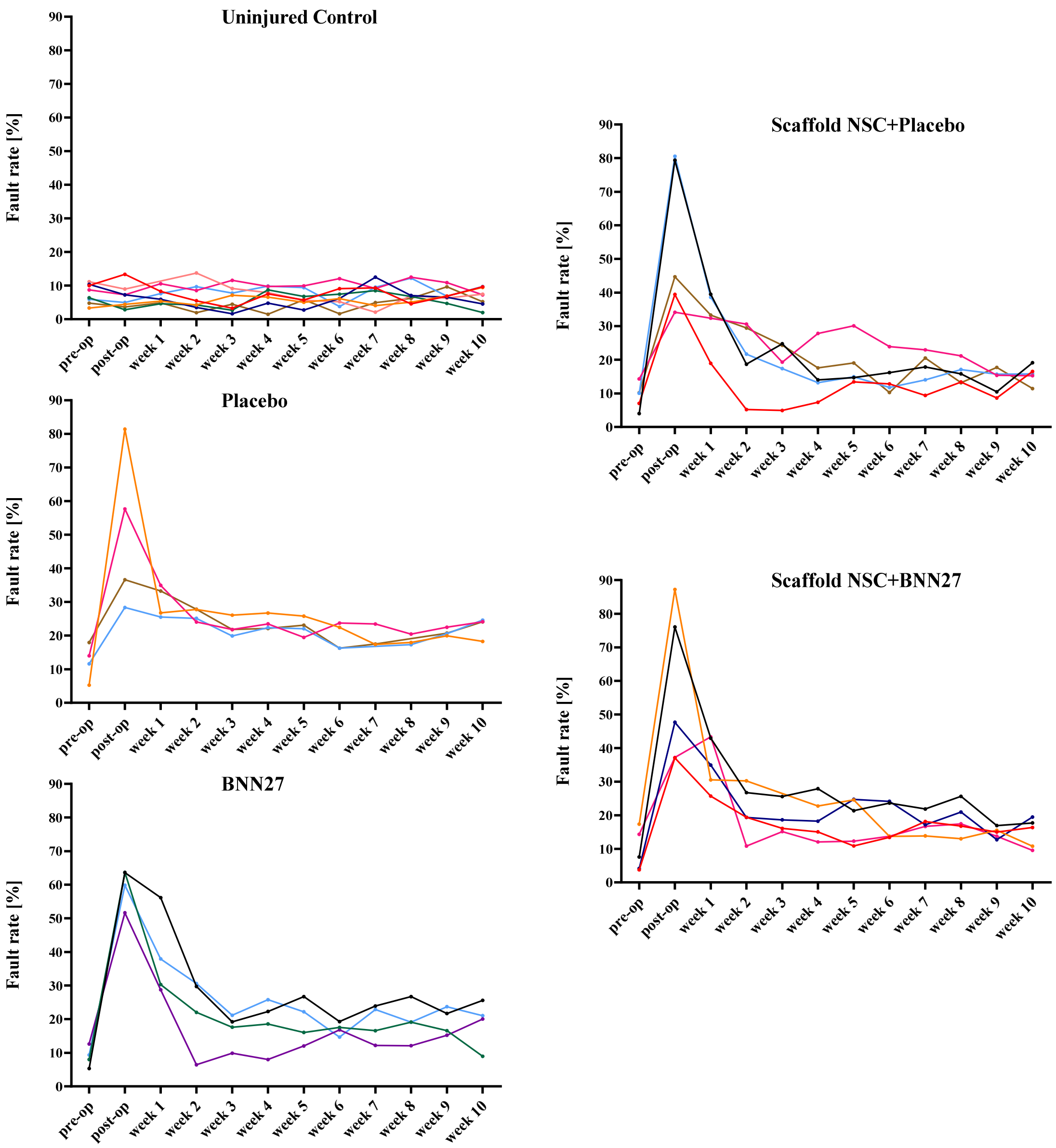

Figure S2. Raw data of the fault rate response in the horizontal ladder walking assay per experimental group. Each curve corresponds to a different animal.

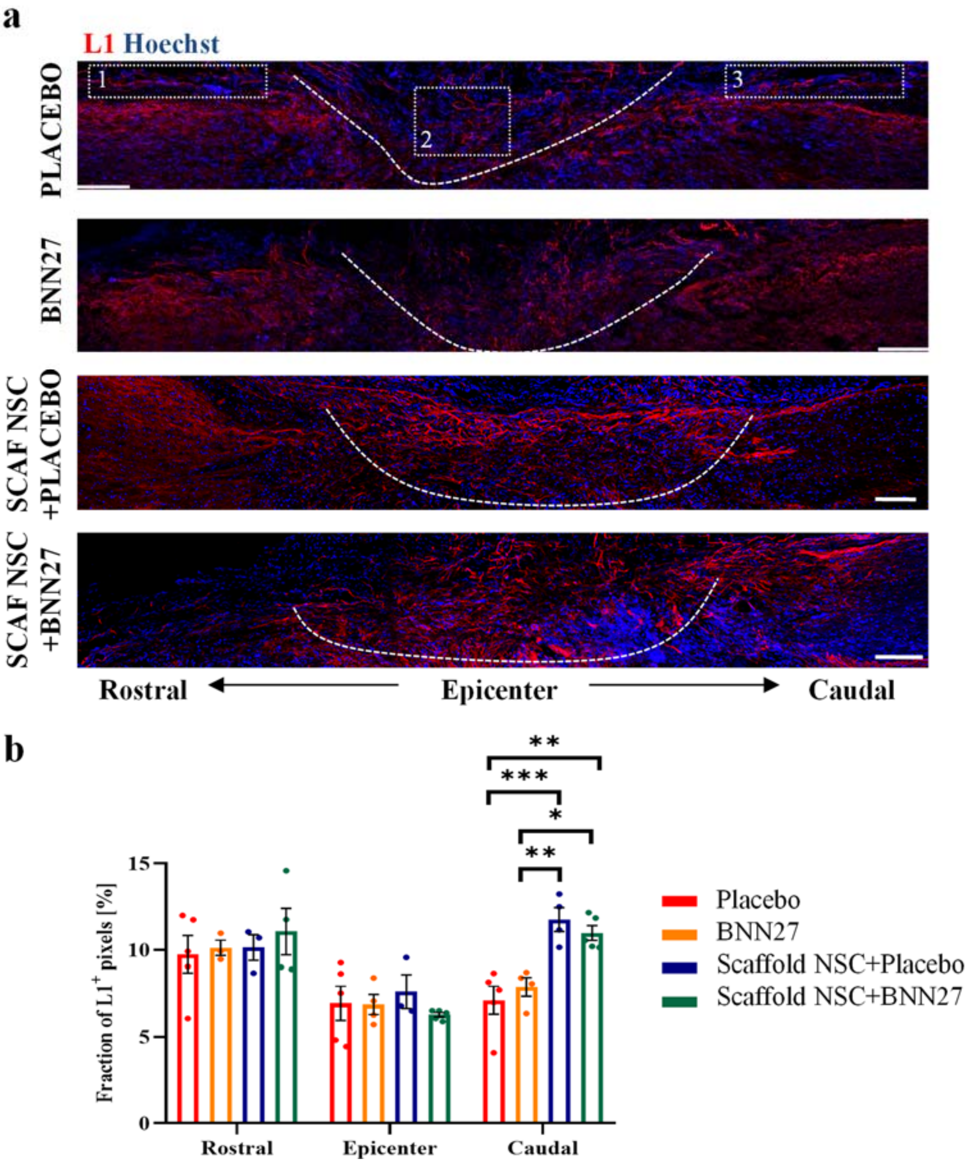

Figure S3. Effects of NSC-seeded PCS on axonal elongation. (a) Representative fluorescence images of spinal cord parasagittal sections in the lesion site from all four injured groups immunostained for L1 (red). The boundaries of lesion site are marked with the dotted line. The dotted squares show the regions that were quantified rostrally (1), in the epicenter (2) and caudally (3) to the lesion. Scale bars: 100 μm. **(b)** Quantification of $L1^+$ pixel fraction at the lesion epicenter, rostrally and caudally to the lesion in the four injured groups at 12 wpi ("Placebo": n=5, "BNN27": n=4, "Scaffold NSC+Placebo": n=4, "Scaffold NSC+BNN27" n=5). Results are presented as mean ± SEM. *P<0.05, **P<0.01, ***P<0.001; Tukey's post-hoc pairwise test assuming $P_{2\text{-}way\text{-}ANOVA}<0.05$.
